# Hepatic Langerhans Cell Histiocytosis (LCH) Presenting as a Harbinger of Multisystem LCH

**DOI:** 10.7759/cureus.8591

**Published:** 2020-06-13

**Authors:** Hua Li, Peter Ells, Mustafa Erdem Arslan, Karl A Robstad, Hwajeong Lee

**Affiliations:** 1 Pathology and Laboratory Medicine, Albany Medical Center, Albany, USA; 2 Gastroenterology, Albany Medical Center, Albany, USA; 3 Pathology and Laboratory Medicine, Columbia Memorial Health, Hudson, USA

**Keywords:** langerhans, liver, histiocytosis, cholangitis

## Abstract

Langerhans cell histiocytosis (LCH) is a rare systemic disorder characterized by an infiltration of CD1a+/langerin+ histiocytes, commonly involving bone, skin, and lymph nodes in children. Hepatic involvement is rarely observed in multisystem LCH. We describe an exceptional case of hepatic LCH in an adult preceding the diagnosis of multisystem LCH, mimicking anti-mitochondrial antibody (AMA)-negative primary biliary cholangitis (PBC). A 65-year-old man presented with intermittent pruritus, weakness, dyspnea, fever, and chills that have been progressive for four years. Physical examination was unremarkable. Laboratory work revealed cholestatic biochemistry profile. Liver biopsy showed portal non-necrotizing granuloma encasing a damaged duct (florid duct lesion), and multifocal lobular Kupffer cell clusters, suggestive of PBC. Tests for autoimmune diseases including AMA were negative. Endoscopic retrograde cholangiopancreatography (ERCP) was negative for biliary obstruction. One month after the liver biopsy, he developed flaky, red, and burning rash on the right scalp, forehead, and epigastric skin. A skin biopsy at an outside institution revealed LCH. Subsequent re-examination of the liver biopsy showed that the histiocytes within the florid duct lesion were positive for CD1a and S-100. Concurrently, a small focus of LCH was noted in his gastric biopsy performed for gastritis symptoms. Hepatic LCH may mimic AMA-negative PBC histologically and clinically and may present as a harbinger of multisystem LCH. While rendering the diagnosis would be challenging without prior history of LCH and with focal involvement, awareness of such presentation and communication with clinical colleagues may be helpful.

## Introduction

Langerhans cell histiocytosis (LCH) is a rare clonal neoplastic proliferation of histiocytes that express CD1a, langerin (CD207+), and S-100 protein. The annual incidence is about five cases per 1 million population that mainly occurs in children [1]. Any organs can be affected alone or in combination, but frequently occurs in skin, bone, and pituitary gland. In 15%-20% of cases, LCH affects spleen, liver, and bone marrow; damage to these organs may be life-threatening [2]. Its clinical presentations are variable, ranging from a single indolent lesion to an explosive multisystem disease [2]. We describe an exceptional case of hepatic LCH in an adult preceding the diagnosis of multisystem LCH, mimicking anti-mitochondrial antibody (AMA)-negative primary biliary cholangitis (PBC) on liver biopsy. The results of this report have been partially presented at the American Society for Clinical Pathology (ASCP) annual meeting in 2019 [3].

## Case presentation

Anonymous case reports are exempt category reviews by the institutional review board (IRB) at the Albany Medical Center, Albany, NY, USA. Written informed consent was obtained from the patient regarding the current case study. 

A 65-year-old man presented with intermittent pruritus, weakness, dyspnea, fever, and chills that have been progressive for four years. Electrocardiogram (EKG), stress test, cardiac catheterization, chest X-ray, coronary computed tomography angiogram (CTA), spirometry, and autoimmune disease workup all turned out negative. Physical examination was unremarkable. Laboratory work for the period from one month before his biopsy to two weeks after revealed elevated alkaline phosphatase (ALP) ranging from 388 to 471 U/L (reference 40-120) on three occasions. His alanine transaminase (ALT) ranged from 31 to 111 U/L (reference 0-40), aspartate aminotransferase (AST) 38-81 U/L (reference 0-40), and bilirubin 0.6-2.0 mg/dL (reference 0-1.2). His gamma glutamyl transpeptidase (GGT) was 271 U/L (reference 0-41) on a single occasion.

**Figure 1:**
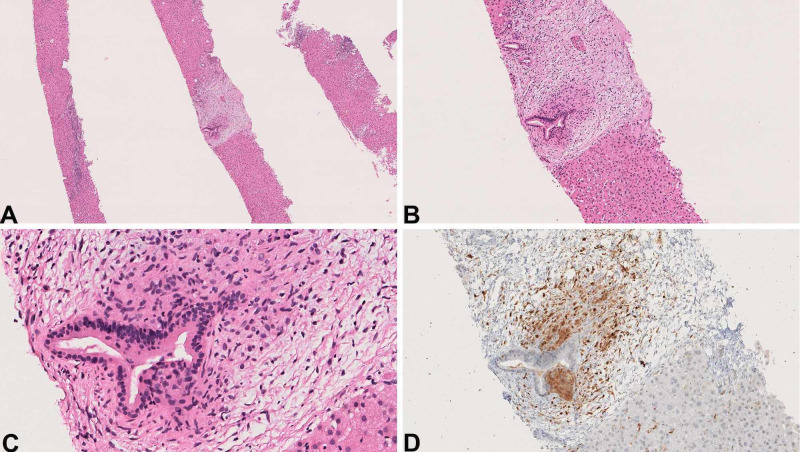
Hepatic LCH mimicking primary biliary cholangitis. (A-C) Lobular and portal non-necrotizing granulomatous inflammation with one “florid duct lesion” (A. Hematoxylin and eosin (H&E), 40x; B. H&E, 100x and C. H&E, 400x). (D) CD1a immunostain highlights Langerhans cells encasing the duct (CD1a, 200x). LCH, Langerhans cell histiocytosis

Liver biopsy showed mild portal inflammatory infiltrate consisting of lymphocytes, plasma cells, and rare eosinophils with no significant interface activity. There was a histiocytic cluster (granuloma) surrounding medium-sized interlobular bile duct associated with duct injury (Figure 1A-C).

The lobules showed frequent Kupffer cell clusters, occasional apoptotic bodies and inflammatory foci. Although the differential diagnoses for hepatic nonnecrotizing granuloma are broad, granuloma-encasing damaged duct (florid duct lesion) in the setting of cholestatic pattern biochemistry is suggestive of PBC. ERCP was negative for biliary obstruction. There was no drug history that would account for cholestatic biochemistry. Given the negative test results, including a negative anti-mitochondrial antibody (AMA), a diagnosis of AMA-negative PBC was considered.

**Figure 2:**
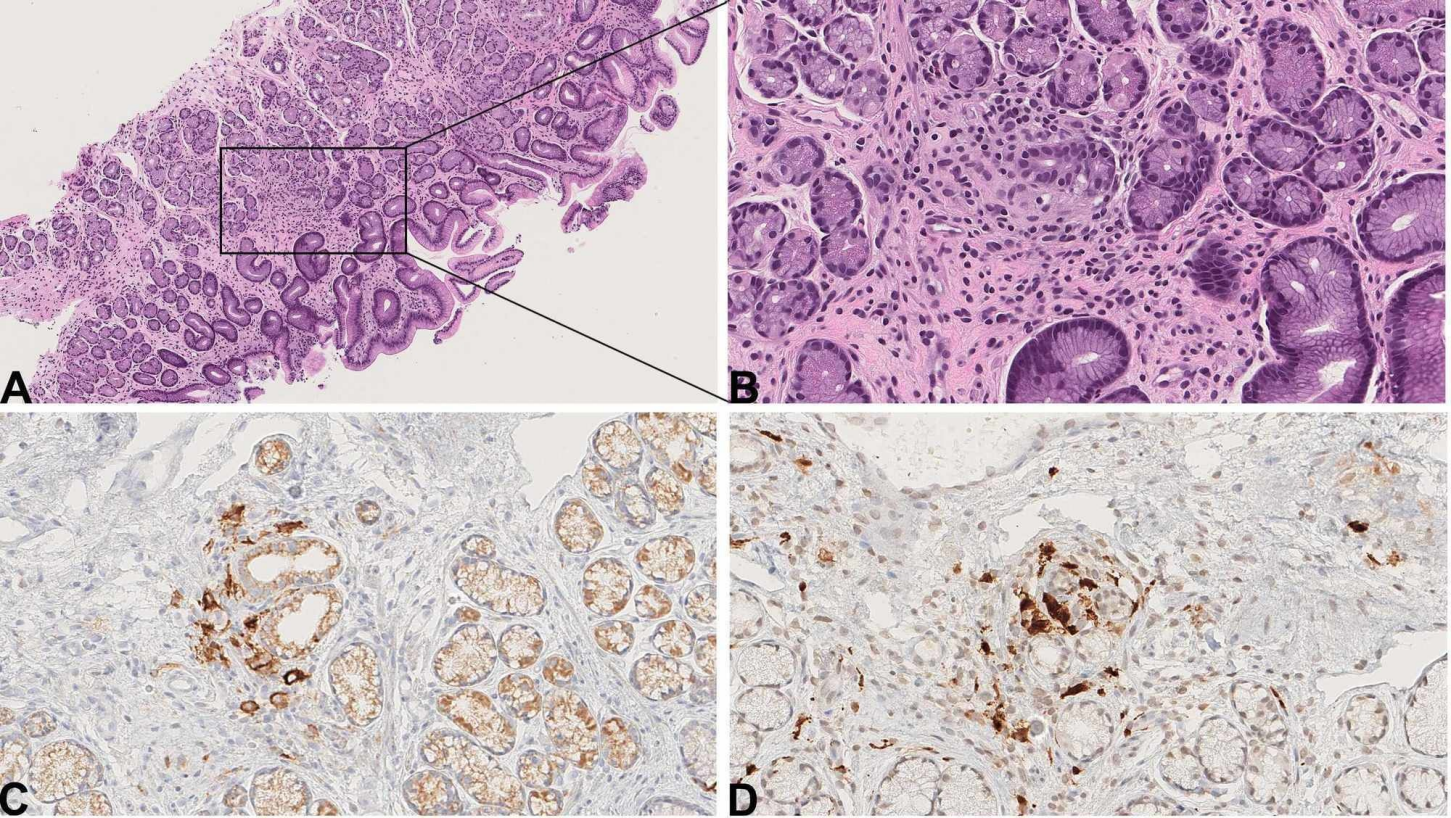
Gastric LCH. (A, B) Focal gastric glands infiltrated by mixed inflammatory cells (A. Hematoxylin and eosin (H&E), 100x; B. H&E, 400x). (C) CD1a and (D) S-100 immunostain highlights Langerhans cells (C. CD1a and D. S-100, 400x). LCH, Langerhans cell histiocytosis

One month after the liver biopsy, the patient developed flaky, red, and burning rash on the right scalp, forehead, and epigastric skin. A skin biopsy at an outside institution revealed dermal and epidermal infiltration of CD1a positive histiocytes with indented nuclei and pale eosinophilic cytoplasm, consistent with LCH. Subsequent re-examination of the liver biopsy showed that the histiocytes surrounding one medium-sized duct, associated with duct injury, were positive for CD1a (Figure 1D) and S-100. In retrospect, rare histiocytes showed equivocal nuclear groove-like structure. However, still, it would have been extremely challenging or nearly impossible to differentiate between PBC-associated granuloma and Langerhans cell cluster based on histomorphology alone. The lobular Kupffer cell clusters were negative for both. Meanwhile, the patient underwent an endoscopic ultrasound to evaluate the bile ducts. This showed “edematous” mucosa in the stomach; a biopsy was obtained. A small focus of LCH was noted in his gastric biopsy (Figure 2A-D).

The patient had a total serum bile acid level of 143.1 µmol/L (reference 3.8-20.9). He was given ursodeoxycholic acid for cholestasis and vemurafenib and prednisone for LCH. Six months later, his symptoms subsided except pruritus. His alkaline phosphatase (ALP) decreased to 244 U/L, GGT decreased to 77 U/L, and bilirubin returned to normal range (total bilirubin 1.0 mg/dL, direct bilirubin 0.3 mg/dL, indirect bilirubin 0.7 mg/dL). At one-year follow up, the patient is doing reasonably well; however, pruritus and cholestatic biochemical profile persisted (GGT 569 U/L, total bilirubin 2.6 mg/dL, direct bilirubin 1.2 mg/dL, and indirect bilirubin 1.4 mg/dL, total bile acid level 169.3 µmol/L).

## Discussion

Langerhans cell histiocytosis (LCH) is a group of disorders characterized by a clonal proliferation of histiocytic/dendritic cells with infiltration by other inflammatory cells, including eosinophils, neutrophils, and small lymphocytes in variable organs. The histiocytes are generally large, round to oval in shape, with a coffee-bean nuclear grove, fine chromatin, and thin nuclear membranes. Diagnostic immunohistochemical markers include S-100 protein, CD1a, and langerin (CD207) [1].

The annual incidence of LCH is about five cases per 1 million population, with most cases occurring in childhood [1]. The incidence of LCH in adults is 10 times less than that in children. In adults, LCH usually presents after the fourth decade. Any organs or systems can be affected; frequently affected organs are the skeleton (80%), skin (33%), and pituitary (25%) [4]. Patients with LCH involving the liver, spleen, or bone marrow are at the highest risk of death from LCH and are therefore classified as having high-risk LCH [2]. The clinical presentation of LCH varies from a solitary eosinophilic granuloma to widespread disseminated disease with organ dysfunction [2].

Hepatic LCH in adults is very rare and is usually restricted to patients with disseminated disease. Patients with hepatic LCH may present with fatigue, pruritus, and jaundice. In some cases, imaging or biochemical studies done for other reasons may show liver abnormalities. Biochemistry studies are significant for elevations of GGT, ALP, and bilirubin, while aminotransferases are usually within normal limits [5-6]. Imaging studies of liver may show solitary or multiple nodular lesions of low densities [5], diffuse hepatomegaly, periportal signal abnormalities, and dilation of biliary ducts and wall thickening [6].

On liver biopsy, two major histological patterns may be noted, alone or in combination. At an early stage of disease, Langerhans cells cluster in the lobules and portal tracts, the extent of which ranges from small granulomatous foci to large tumor-like masses. Langerhans cells are commonly accompanied by a mixture of other inflammatory cells, where eosinophils frequently predominate [7-8]. In portal tract, Langerhans cells may surround and infiltrate the bile ducts, and may cause bile duct injury and proliferation [7]. At the late stage, some severe cases demonstrate periductal fibrosis with little or no histiocytic infiltrates, consistent with secondary sclerosing cholangitis [6]. Clinically patients at this stage manifest with severe cholestasis and liver failure. A subset of patients progresses to develop cirrhosis [8-9].

Rendering a diagnosis of hepatic LCH without clinical suspicion can be extremely challenging. Due to its patchy distribution and limited sampling, there may be little or no CD1a +, langerin (CD207) + histiocytes in the biopsy [7]. If the liver biopsy is done at a late stage of the disease, the biopsy may show fibrosis only with little or no histiocytes [6]. In the latter scenario, even if the clinical history of LCH were to be provided, it would be very difficult to differentiate secondary sclerosing cholangitis associated with LCH from primary sclerosing cholangitis. To make this distinction, the entire biopsy has to be carefully examined for evidence of LCH and a “beaded” appearance of extrahepatic biliary tree by imaging may be helpful for the diagnosis of primary sclerosing cholangitis.

As demonstrated in the current case, it would be difficult to differentiate hepatic Langerhans cell clusters from PBC-associated granulomas in a small liver biopsy. Lack of usual histological features of PBC, such as dense portal lymphocytic infiltrate and mild degree of interface activity, and AMA negativity might be a clue. In addition, recognizing nuclear grooves of Langerhans cells might be helpful. However, this distinction will likely remain challenging when the clinical history of LCH is not provided and the involvement is focal.

Treatment strategy for LCH varies depending on the degree of organ involvement and clinical course. For patients with solitary disease, only local therapy or observation is usually required. Multisystem LCH requires systemic steroid and chemotherapy, such as vinblastine, methotrexate, and cladribine [9]. The prognosis of patients with hepatic LCH is worse than those with nonhepatic LCH, with a fatality rate of 30% versus 10%. Early stage disease with either nodules or hepatomegaly due to histiocytic infiltration responds well to chemotherapy. However, late stage disease with either cirrhosis or liver insufficiency is difficult to treat [9]. Thus, early diagnosis is important.

Herein we report a case of an adult with hepatic LCH preceding signs of multisystem LCH. The patient presented with nonspecific symptoms (e.g. pruritus, weakness, dyspnea, fever, and chills), cholestatic biochemical profile, and negative imaging studies. Liver biopsy showed portal chronic inflammatory infiltration with a focus of granuloma surrounding a medium-sized bile duct (mimicking florid duct lesion) associated with duct injury. In addition, multiple lobular Kupffer cell clusters were noted. Florid duct lesion and cholestatic biochemical profile strongly suggested AMA-negative PBC or a drug-induced cholestatic liver injury, which are more common than hepatic LCH. Subsequent diagnosis of cutaneous LCH in this patient made us consider the possibility of hepatic involvement of multisystem LCH. Re-examination of the liver biopsy and concurrent gastric biopsy finally confirmed the diagnosis of LCH involving these organs.

## Conclusions

Hepatic LCH may present as a harbinger of multisystem LCH. Moreover, hepatic LCH may mimic AMA-negative PBC histologically and clinically. Rendering the diagnosis of hepatic LCH would be challenging without prior history of LCH and with focal involvement. However, awareness of such presentation and communication with clinical colleagues may be helpful.
